# A New Direction in Microfluidics: Printed Porous Materials

**DOI:** 10.3390/mi12060671

**Published:** 2021-06-08

**Authors:** Hanno Evard, Hans Priks, Indrek Saar, Heili Aavola, Tarmo Tamm, Ivo Leito

**Affiliations:** 1 Institute of Chemistry, Chair of Analytical Chemistry, University of Tartu, Ravila 14a, 50411 Tartu, Estonia; indrek.saar@ut.ee (I.S.); heili.aavola@gmail.com (H.A.); ivo.leito@ut.ee (I.L.); 2 Intelligent Materials and Systems Lab, Institute of Technology, University of Tartu, Nooruse 1, 50411 Tartu, Estonia; m6te@live.com (H.P.); tarmo.tamm@ut.ee (T.T.)

**Keywords:** microfluidics, porous material microfluidics, screen printing, direct write printing, photolithography

## Abstract

In this work, the feasibility of a novel direction for microfluidics is studied by demonstrating a set of new methods to fabricate microfluidic systems. Similarly to microfluidic paper-based analytical devices, porous materials are being used. However, alternative porous materials and different printing methods are used here to give the material the necessary pattern to act as a microfluidic system. In this work, microfluidic systems were produced by the following three separate methods: (1) by curing a porous monolithic polymer sheet into a necessary pattern with photolithography, (2) by screen printing silica gel particles with gypsum, and (3) by dispensing silica gel particles with polyvinyl acetate binder using a modified 3D printer. Different parameters of the printed chips were determined (strength of the printed material, printing accuracy, printed material height, wetting characteristics, repeatability) to evaluate whether the printed chips were suitable for use in microfluidics. All three approaches were found to be suitable, and therefore the novel approach to microfluidics was successfully demonstrated.

## 1. Introduction

Over the past 30 years, microfluidics has emerged as a promising technological solution for problems in numerous fields [1,2], including, prominently, analytical chemistry. One of the aims of this field is to develop a micro total analysis system that would enable carrying out the whole chemical analysis on a single chip (“Sample in, answer out”) [2]. Although this goal has been occasionally achieved in laboratory conditions [3,4,5], as of now only a few microfluidic systems have gained commercial success [6,7]. The main challenge has been achieving low cost and high performance at the same time [8]. External devices (e.g., pumps and detectors) must often be used together with the microfluidic system to achieve the necessary functionality or analytical performance, leading to high cost [6,9]. It is also difficult to achieve integration of different materials into a functioning device in a cost-effective manner [8,10].

An alternative approach to microfluidics, called microfluidic paper-based analytical devices (μPAD), has emerged in the last 10 years [11]. Compared to the usual solid materials, where channels must be formed, the paper already has a porous structure, and therefore there is no need to create any microscale channels. It is only necessary to produce barriers in the paper to direct the flow. This can be achieved with the following three different methods: (1) cutting out a specific pattern from the paper, (2) filling the pores of the paper in specific areas, or (3) making the surface of the paper nonpolar in specific areas [12,13,14]. Some of these methods—e.g., wax printing—are simple, cheap and scalable to mass production [15,16]. The hydrophilic nature of paper allows liquids to move in the paper structure by capillary forces, and therefore external pumps are not necessary. Moreover, detection methods are simple and well known—paper and similar materials have been used in analytical chemistry for over 40 years for different tests (indicator paper, pregnancy tests, etc.). As a result, μPAD’s have been widely applied to a wide range of fields for chemical analysis, such as clinical analysis (especially in point-of-care settings [7,17]), food and water analysis [18], environmental analysis [19], forensics [20], etc.

However, paper presents several problems when used as the substrate for microfluidics. Having good control over the surface chemistry, pore size distribution and thickness of the material is important for microfluidics, as these parameters determine the flow characteristics of liquids in the porous material [7,21]. The possibilities of accurate control of liquid flow in the paper are limited at the moment [22]. The surface of common filter paper is covered with hydroxy groups, which are relatively unreactive and therefore need harsh chemicals to be modified [23]. The porosity and thickness of papers cannot be totally controlled, depending on commercially available materials. An additional problem with paper is that different analytical steps (e.g., sample preparation steps like solid-phase extraction and chromatography) are difficult to achieve on a single device, as these steps demand different surface chemistry and porosity for optimal performance.

A solution for this would be a technology that allows a wider choice of porous materials to be patterned. Different printing methods have been considered for this, but in a limited scope. In one case, a 3D printer was modified for printing a slurry of thin-layer chromatography (TLC) materials to produce the porous material in any necessary pattern [24], but microfluidics was not considered as a possible application. Producing a mesoporous material for microfluidics with sol-gel and different printing methods has been also demonstrated [25]; however, in this case only mesoporous materials are produced (common porous materials used in analytical chemistry are made of particles, and therefore also have macropores between the particles, e.g., TLC plates, solid-phase extraction columns, etc.).

It must also be noted that the direction demonstrated in this article is distinctly different from producing a porous material with 3D printing, e.g., for microchip liquid chromatography [26], or using photolithography to produce micropillar array columns [27]. In those cases, the material is produced in a systematic pattern and there is still a need for highly accurate production methods, similar to the methods where precise microfluidic channels are formed into a solid. 

The goal of this work was to demonstrate the following new direction in microfluidics: printing methods can be used to produce patterns of porous materials for microfluidics. Three printing methods were used to demonstrate this. First, photolithography (PL) was used to produce porous polymer sheets in the necessary pattern. Second and third, screen printing (SP) and direct write printing (DWP), respectively, were demonstrated as a possible techniques to print microparticles with a binder.

## 2. Materials and Methods

### 2.1. Photolithography

Trimethylolpropane tris(3-mercaptopropionate) (Sigma-Aldrich, St. Louis, MO, USA) and triallyl-s-triazine-2,4,6(1H,3H,5H)-trione (Acros) were used as monomers to produce a polymer by thiol–ene click chemistry. Methanol (Honeywell, CHROMSOLV) was used as a porogen and azobisisobutyronitrile (Sigma-Aldrich) as the initiator for polymerization. The initiator was first dissolved in methanol (25 mg per 1 mL of the whole prepolymer mixture) after which monomers were added and the liquid was thoroughly mixed. The prepolymer solution consisted of 60% v/v methanol. The amounts of monomers were chosen so that the ratio of the thiol and ene functional groups would be 1:1. Care was taken to keep the mixture in the dark during preparation and the mixture was used immediately after preparation.

Polytetrafluoroethylene tape was placed as a spacer between two polysine microscope slides (Thermo Scientific, Waltham, MA, USA). The distance between the slides was measured to be 0.4 mm. The bottom slide was covered with Parafilm to promote the synthesized monolith to stick only to the top slide. For PL, a mask was printed onto a transparent film with a laser printer (Canon LBP6230, Canon Inc., Tokyo, Japan). The mask was then placed on top of the microscope slide. The mask and the slides were held in place by binder clips. The prepolymer mixture was then injected between two microscope slides from a syringe so that the whole space between the two slides would contain the prepolymer.

The slides were placed approximately 10 mm from a UV lamp for curing (a 15 W lamp that provides light at 365 nm was used). Curing time depended on the experiments and is discussed in the results section. After curing the polymer, the slides were carefully taken apart, uncured prepolymer was discarded and the cured material was washed carefully several times with acetone to remove all the initiator and monomers from the pores of the monolith.

Pictures of the setup can be found in electronic Supplementary Information (ESI Figures S1 and S2).

### 2.2. Screen Printing

A polyester screen with T8 mesh and thread diameter of 300 micrometres on a wooden frame was used for SP.

The mask for the stencil was printed onto a transparent film with a laser printer (Canon LBP6230). The stencil was formed onto the screen with KIWO Azocol Z1 universal emulsion. A 15 W UV lamp was used for curing the emulsion for 3 h. The stencil was produced onto the screen so that the channels of the microfluidic system would be approximately at a 45-degree angle with the threads.

The slurry made for printing consisted of TLC mixture (Sigma-Aldrich, St. Louis, MO, USA, high-purity silica gel (7749) with gypsum and a fluorescent indicator) and deionized water (Millipore, Milli-Q Advantage A10). After thorough stirring of the powder and water, the slurry was left to age for 30 min. Thereafter, the slurry was thoroughly stirred again before printing. The slurry was printed onto a polysine microscope slide. Printing was carried out manually with usual SP techniques. The distance between the screen and the slide was set to approximately 0.25 mm.

### 2.3. Direct Write Printing

An aqueous solution of polyvinyl acetate (PVAc) was prepared by weighing 15.2 g of the glue (Fila, Giotto Vinilink) into a 50 mL volumetric flask and filling the flask to the mark with deionized water. To prepare the slurry, glycerol (Sigma-Aldrich), silica gel microparticles (SG60, 60741, Fluka) and the PVAc solution were thoroughly mixed in a vial (specific ratios discussed below). Thereafter, the slurry was cycled 12 times in vacuum to remove air bubbles. The slurry was carefully mixed again on a magnetic stirrer and poured into the syringe (Adhesive Dispensing, Milton Keynes, UK) for printing.

A modified 3D printer (K8200 Velleman, Velleman, Inc., Gavere, Belgium) applicable for direct pressure dispensation was used in these experiments. G-code of the structure was written and executed with open source 3D printing software (Repetier-Host 2.1.6, Germany), with printing speed of 10 mm s^−1^, layer height of 0.2 mm and a 18G printing nozzle (Adhesive Dispensing, Milton Keynes, UK). A chip design with 3 channels was printed (see Section 3.3 Direct Write Printing). The extrusion multiplier (flow rate) was increased for each consecutive channel. The extrusion multipliers used for the 3 channels were 6×, 10× and 14×. The structures were printed onto polysine microscope slides and after printing, the slides were placed into an oven at 75 °C until glycerol had evaporated (about 5 days).

### 2.4. Instrumentation

A Leica optical microscope (M165 FC) with 7.3× magnification and Leica Application Suite (version 3.7.0) analysis tool was used to measure the width of the formed channels. The measurements were taken from 6 different locations of the channel so that the results would have as high variability as possible (i.e., widest and narrowest spots were chosen).

The thickness of the printed material was measured by placing a glass slide on top of the printed material and measuring the thickness with a 0.01 mm resolution calliper. The thickness of two glass slides was subtracted from the result to obtain the thickness of the material.

As commonly done with nitrocellulose membranes [28], wetting time (i.e., time that is needed to wet 2 cm of the material) was used to measure the flow characteristics of the materials. The measurements were made by immersing approximately 5 mm of the bottom rectangular-shaped area into an aqueous solution of a colourant (food colouring containing brilliant blue FCF (acquired from local store) in case of PL and DWP, and xylenol orange (Reanal, Budapest, Hungary) in case of SP). A millimetre paper was placed on the back of the glass slide and pictures were taken with a camera as the material wetted. The length of wetted material as a function of time was recorded, and the approximate wetting times were calculated (in units of s/2 cm). Whatman grade 1 (GE Healthcare, Chicago, IL, USA) filter paper was cut to a similar pattern as the printed materials with Cricut Joy (Cricut, Inc., South Jordan, UT, USA) cutter-plotter to measure wetting times in comparison with the printed materials (the channel widths of the patterned filter paper were 1.8, 2.1 and 2.4 mm).

The samples of materials were dried in a vacuum chamber (pressure until 2 mBar), coated with 7 nm of gold (EM ACE600, Leica, Wetzlar, Germany) and imaged with a tabletop scanning electron microscope (SEM) (TM-3000, Hitachi, Tokyo, Japan).

Fujifilm X-A3 camera with XC 16–50 mm objective was used to take photographs of the experiments.

## 3. Results

In this article, the following criteria were set to assess whether a method is suitable for producing a microfluidic system: (1) it should be possible to produce long and narrow (down to 3 mm) “channel” shapes, and larger rectangular shapes of the porous material, (2) the printed material must be strong enough so that it does not break when the substrate is turned upside down or handled during experimentation, (3) the printed material must not come lose when water is added to the material, (4) the microfluidic system must function in a reasonable timeframe, i.e., wetting times should not be prohibitively long (wetting time should be within the same order of magnitude as for Whatman grade 1 filter paper or lower), and (5) it should be possible to meet the above criteria at least four times in five replicates. Moreover, the limit of the smallest channel width was found for each printing method by printing channels with different widths.

### 3.1. Photolithography

**Figure 1:**
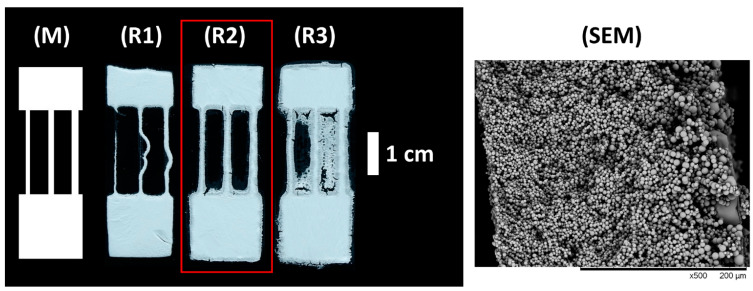
The mask used for PL experiments (M), the resulting pattern of the monolithic thiol–ene polymer sheet with different curing times (**R1**–**R3**), and SEM micrographs of the cross-section of the material. The channel widths of the mask from left to right are as follows: 0.9 mm, 1.2 mm, 1.5 mm. The length of the channels is 2 cm and the length of the whole shape is 4.5 cm. The width of the shape is 1.5 cm. The curing times were 45, 65 and 80 s for (**R1**–**R3**), respectively. The conditions used for print (**R2**), highlighted with the red box, were found to be optimal for PL.

A porous monolithic polymer is formed when polymerization takes place in the presence of a porogen—a solvent that can dissolve the monomers but not the polymer. This approach is widely used to produce monolithic chromatography columns [29]. In this work, PL was used to produce a specifically patterned sheet of porous polymer monolith forming a microfluidic system. The pattern of the mask used for PL can be found in Figure 1 (M).

It was found that optimizing the curing duration is essential to achieve good accuracy (i.e., how well the printed shape resembles the designed shape). The best results were achieved with a curing time of 65 s in the case of this experimental setup (see Figure 1 (R2) and comments in ESI). The SEM micrographs of the monolith material cured for 65 s can be found in Figure 1 (SEM) and ESI Figure S3.

Five replicates were produced with the curing time of 65 s (ESI Figure S4). The measurement data of the material thickness and channel widths can be found in the ESI (Tables S5–S7). During curing, widening of the channels had occurred when compared to the widths of the channels on the mask. The reasons for this loss of accuracy can happen due to UV radiation passing through the mask, light passing under the mask as collimated light was not used, and diffusion of reactive free radicals created during curing [30].

For the narrowest channels (and, in some cases, other areas of the material) some of the following issues occurred: the synthesized material was relatively brittle and can break, and the material can delaminate from the glass slide leading to curved channel shapes. Due to these reasons, the narrowest channel could not be produced properly in one of the replicates (ESI Figure S4). Therefore, these channels were considered too narrow for this printing procedure. Thiol–ene chemistry allows covalent bonding to glass [31], which can lead to a durable porous material that does not break or delaminate easily.

The porous material does not have a polar surface, and therefore does not wet with water immediately after synthesis. To achieve wetting of the material, it was left exposed to air at room temperature for at least 3 days. As a result, the wetting properties of the material changed, and the porous structure could be easily wetted with water. Although establishing the specific reason for this is outside the scope of this study, it is reasonable to expect that oxidation leads to the formation of polar functional groups on the surface of the material. Energy dispersive X-ray spectroscopy analysis (SwiftED-3000, Oxford Scientific) showed that the oxygen percentage in the material changed from 14.9% to 22.3% within one week after synthesis (ESI Table S1).

**Figure 2:**
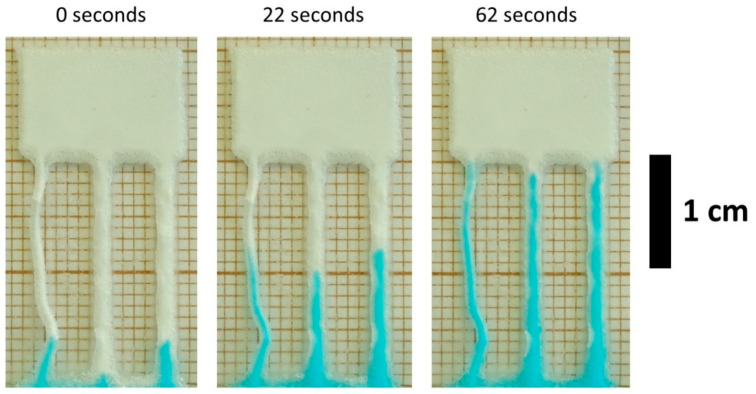
Example of wetting time measurements for material printed with PL method.

The wetting time for the channels was measured 1 week after synthesis (see example in Figure 2). The data on wetting times can be found in ESI (Tables S8 and S9). Generally, it is concluded that PL fills all the criteria set in this article for the microfluidic system (see Table 1).

### 3.2. Screen Printing

SP was used to print TLC stationary phase material to form the microfluidic system.

**Figure 3:**
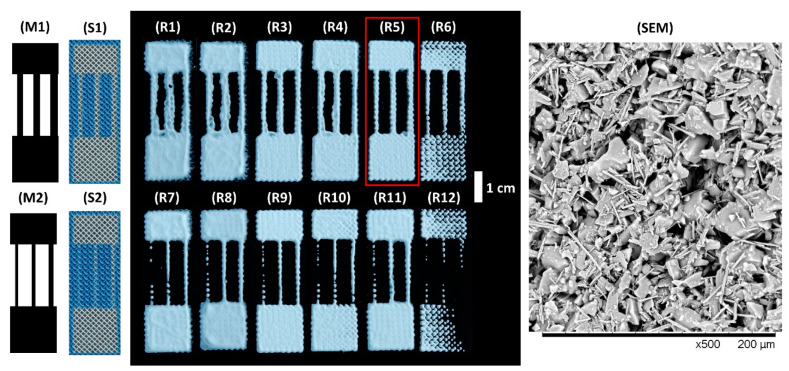
Masks used for preparing the stencil on the SP screen (**M1** and **M2**), the corresponding stencils prepared on the screen (**S1** and **S2**), the results of screen printing with different concentrations of the TLC mixture (**R1**–**R12**), and SEM micrograph of the silica gel microparticles and gypsum mixture. Widths of channels on mask (**M1**) from left to right are as follows: 1.8 mm, 2.1 mm and 2.4 mm. Widths of channels on mask (**M2**) from left to right are as follows: 0.9 mm, 1.2 mm, 1.5 mm. The length of the channels was 2 cm and the length of the whole shape was 4.5 cm. The width of each shape was 1.5 cm. In both rows the slurry concentration increases from left to right, as follows: 0.35, 0.40, 0.43, 0.47, 0.51, 0.57 g/mL. The conditions used for print (**R5**), highlighted with the red box, were found to be optimal.

The masks used to prepare the stencil on the screen can be seen in Figure 3 (M1) and (M2). Different concentrations of the TLC mixture in water (0.35, 0.40, 0.43, 0.47, 0.51, 0.57 g/mL) were tested to find the optimal slurry consistency for printing. The results can be seen in Figure 3. The highest and lowest concentrations were found to be unsuitable due to the unsuitable viscosity of the mixture for printing. When the pattern (S2) was printed, the formation of the channels was inconsistent—i.e., in all cases, gaps were left in the thinnest channels due to the blocked mesh openings in the screen (see Figure 3 (S2)). Narrower stencils can be properly formed on a suitable screen [32].

The higher viscosity slurry left jagged edges for the printed material (R5 and R6 in Figure 3). The reason for this was the large mesh openings of the used screen and the viscosity of the slurry, which does not allow flowing of the ink after printing. Using a screen with smaller mesh openings will lead to less jagged edges. However, in the current work, the shape of the edge did not impede the printed material to be used for microfluidics.

The accuracy of the printed channels (Table S2 in ESI) and thickness of the printed materials (Table S3 in ESI) was measured for the prints (R1) to (R5). It was found that at higher mixture concentrations, the width of the channels became narrower due to increase in the viscosity of the mixture (higher viscosity leads to less flow of ink after printing). A more detailed discussion on the results can be found in ESI.

**Figure 4:**
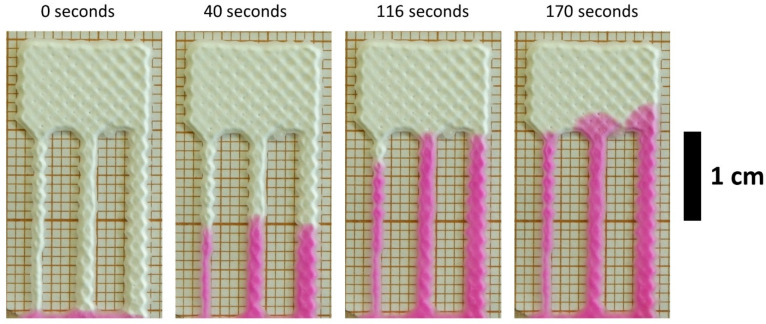
Example of wetting time measurement for one of the replicate printed with screen printing.

The wetting time of the channels was measured and compared for prints (R1)–(R5) (see example in Figure 4 and data in ESI Table S4 and Figure S5). During the wetting test, the submerged part of the material flakes off from the glass. Only at the concentration of 0.51 g/mL the printed material did not come loose (ESI Figure S6). These results can be explained by the fact that the strength of the gypsum binder depends on the water/gypsum ratio—as this ratio decreases, the strength of the gypsum increases [33]. Therefore, the softer material can come off the glass faster during the wetting test, leading to less flow of liquid onto the chip, hence, lowering the wetting time.

From accuracy and wetting time measurements, 0.51 g/mL was found to be optimal for this printing procedure.

The pattern (S1) was printed five times with 0.51 g/mL slurry to demonstrate repeatability. The channel width, material height and wetting times of the replicates can be found in ESI Tables S5–S9. All of the replicates fill the criteria set for the microfluidic systems in this article (see Table 1).

### 3.3. Direct Write Printing

Lastly, an alternative printing method to SP, DWP, was used to make porous microfluidic systems with microparticles and a binder.

**Figure 5:**
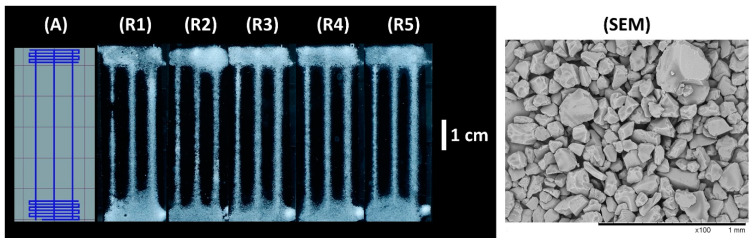
Nozzle movement pattern when printing with DWP (**A**), five replicate prints (**R1**–**R5**), and SEM micrographs of the printed silica gel microparticles with PVAc. The extrusion multipliers used for the three channels were 6×, 10× and 14× from left to right.

First, it was found that a PVAc concentration of 0.015 g/mL was suitable as the particles were strongly bound together and the binder did not interfere with the wetting of the formed porous material. The printing was tested at different concentration levels of microparticles in the slurry, and 0.3 g/mL was found to be optimal as higher concentrations had too high viscous for printing. Five replicate chips were printed (see results in Figure 5).

**Figure 6:**
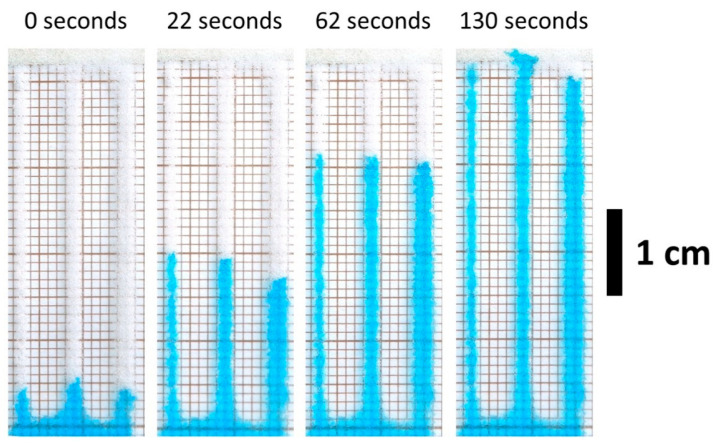
Example of wetting time measurement with print (**R5**) from Figure 5, printed with DWP.

The wetting times were measured on four chips (see example in Figure 6). The measurement results can be seen in Tables S8 and S9 in ESI. The narrowest channel of R1 did not wet, and all three channels of R2 did not wet due to gaps (i.e., large pore size) in the channels (see ESI Figures S9 and S10). The SEM micrographs of the silica gel particles (ESI Figures S11 and S12) reveal that the particles were irregularly shaped and range from approximately 66 to 310 μm in size, which caused this phenomenon [34]. Therefore, it is expected that using smaller, spherically shaped particles will alleviate these issues. Also, printing the material on the same area more than once can resolve this problem (ESI Figure S13).

In DWP, a further increase in accuracy can be achieved by using a printer that allows retraction of the plunger during nozzle movement, where printing is not performed. As a result, the ink would not keep flowing during non-print moves of the nozzle, and the appropriate amount of ink will be dispensed when the printing is continued.

The widths of the channels and thickness of the material of the prints (R1)–(R5) were measured (ESI Tables S5–S7). Similarly to the other printing methods, DWP also fills the criteria set for the microfluidic systems (see Table 1).

## 4. Discussion and Comparison of the Methods

All three printing methods fill the following criteria set for microfluidic systems in this article: narrow “channels” and wider material areas can be printed, sufficient strength of the printed material can be achieved and the materials can withstand wetting with water, the wetting times of the material were not prohibitively long (overall average wetting time for filter paper is higher than for the printed materials, see Table 1 and data in ESI Tables S8 and S9), and the repeatability of production was acceptable for the proof-of-concept stage. The overall comparison of the printing methods to the criteria can be found in Table 1. The tables comparing the measured wetting times, widths of channels and material thickness can be found in ESI (Tables S5–S9).

It was found that the smallest suitable channel widths were 2.1, 2.7 and 2.3 mm for PL, SP and DWP, respectively. These channel widths are clearly suitable for producing a microfluidics system, as these values are within the same range as the channel widths produced in μPAD’s [36], and are below the set 3 mm limit. The narrower channels had different issues (delamination or inaccurate and defective printing), which must be solved in further research.

The accuracy of the printing can be compared only for PL and SP, since no specific channel width could be designed beforehand in DWP. All the measurement results can be seen in Table S7 in ESI. Both PL and SP produce wider channels than designed. The better accuracy values in SP for the narrower channel are explained by the viscosity increase during the printing (see Section 3.2); however, in the case of PL, the reason for the better accuracy of the narrowest channel is unclear.

Although the thickness of the material was found to be relatively similar in the case of all three of the printing methods, the pore sizes of the materials vary greatly between the methods. This is because the size and shape of the particles that make up the porous materials varies between the methods. Moreover, the surface chemistry is also different (for SP and DWP the surface chemistry also depends somewhat on the used binder), and therefore the wetting times of the materials are different. The importance of surface chemistry on wetting time was clearly seen in the case of PL and DWP, as follows: a greater oxidation percent leads to lower wetting times in PL, and using more PVAc leads to higher wetting times (due to the non-polar properties of the binder) in DWP.

To compare the printing methods, the price of the chip, the simplicity of prototyping and transferring the manufacturing to large scale must be considered. The following approximate price of the materials per chip were calculated for each printing method: €0.21, €0.03 and €0.01 for PL, SP and DWP, respectively. As an example, in the case of μPAD’s, it has been calculated that a single device can cost below $0.05 [13]. Compared to PL and DWP, SP has the advantage that it is clearly suitable for high-volume production, which is imperative for commercialization [6]. On the other hand, DWP allows quick prototyping of the microfluidic chips, as only a digital design of the chip is necessary (no masks are needed). In the case of μPAD’s, wax printing, for example, has been shown for prototyping and batch fabrication [16].

### Future Outlook

The general vision of this novel microfluidics direction is that (1) it would be possible to control surface chemistry, material thickness and porosity at different locations of the chip, (2) the chip (i.e., the materials and production) would be cheap. This would lead to the possibility to develop low-cost chips with a high degree of automation and high analytical performance, as optimal materials can be used in different steps of the analysis (e.g., sample preparation by solid-phase extraction or chromatography). These characteristics will allow the chips to be applied to the same problems as have been envisioned for μPAD’s, and in applications where μPAD’s capabilities are still lacking [37].

In the case of all three methods, it is reasonable to expect that the thickness of the material can be manipulated by changing different parameters (distance between glass plates in PL, gap distance or printing several layers of the material on the same area in SP and extrusion rate in DWP). Moreover, surface chemistry and porosity can be easily modified by changing the particles used with DWP and SP. In the case of DWP and SP, combining different materials on the same chip will be possible through printing different parts of the chip with different particles. For PL, changing the composition of the prepolymer can control the porosity, and the surface chemistry can be modified through the active functional groups of the monomers on the surface of the formed polymer [29,38]. Since the thiol–ene reaction is initiated by UV light, photolithography can also be used to modify the surface of the material only at certain places [39], and therefore the exact surface modification of different areas is achievable. In general, porous monolithic materials can be prepared from a wide range of precursors and additives [40]. Therefore, PL is expected to have a broad choice of possible materials (and therefore functionalities) that can be achieved on the microfluidic chips.

## 5. Conclusions

The following novel direction in microfluidics is introduced in this work: the printing of porous materials with predefined patterns for microfluidics. The applicability of three different printing methods was successfully demonstrated in this article. Photolithography was used to make the microfluidic system from a sheet of porous polymer monolith. Screen printing and direct-write printing with a modified 3D printer were used to make the microfluidic system from microparticles and a binder.

All the printing methods were capable of fulfilling the following criteria set in this paper (see Section 3): narrow “channel” shapes and wider areas of material can be printed, the printed materials have suitable strength and can withstand wetting with water, the wetting time of the printed materials is suitable, and the repeatability of producing the microfluidic system is within the set limits.

In the following research, further improvements will be carried out to achieve higher accuracy and repeatability. The control of surface chemistry and porosity will be demonstrated, and chemical analysis applications will be developed. Furthermore, it is the authors’ understanding that multiple other printing methods and materials could be explored to produce microfluidic systems within this new direction.

## Figures and Tables

**Table 1:** Comparison of the different printing methods to (1) the criteria set in this article to the microfluidic system and (2) each other.

Criteria	Photolithography	Screen Printing	Direct Write Printing
Narrowest channel width suitable for microfluidics	2.1 (8% RSD) mm	2.7 (13% RSD) mm	2.3 (16% RSD) mm
Printed material strong enough to be handled	Yes	Yes	Yes
Printed material can withstand being wetted with water	Yes	Yes	Yes
Printed material overall average wetting time ^1^ (compared to filter paper: 206.9 (12% RSD) s/2 cm)	61.1 s/2 cm (44% RSD)	116.3 s/2 cm (5% RSD)	35.3 s/2 cm (22% RSD)
Number of replicates where criteria were met (from 5 replicates in total) ^2^	5	5	4
Average measured thickness of the printed material (thickness of filter paper: 180 μm) ^3^	0.31 mm (6.8% RSD)	0.37 mm (3.8% RSD)	0.33 mm (14.8% RSD)
Pore sizes, measured from SEM images (pore size of filter paper: 11 μm) ^3^	1–9 μm	3–36 μm	34–215 μm
Print accuracy of widest channel (average width subtracted from designed width)	0.87 mm	1.09 mm	–

^1^ Average wetting times and RSD are calculated from channels with widths found to be suitable for microfluidics (i.e., 2.1 mm and higher for PL, 2.7 mm and higher for SP, 2.3 mm and higher for DWP). ^2^ The narrow channels, which were found to be out of the limits for these printing methods, are not taken into account when considering whether the microfluidic chip fills the criteria. ^3^ Characteristics of Whatman grade 1 filter paper taken from [35].

## Supplementary Materials

The Supplementary Materials are available online at https://www.mdpi.com/article/10.3390/mi12060671/s1. Figure S1: Side view of scheme of photolithography setup. The different layers are held together by paper clamps, Figure S2: Pictures of photolithography setup. Figure S3: SEM micrographs of the porous structure of the monolithic thiol-ene polymer sheet. Micrograph (a) shows the cross section of the polymer sheet, (b) shows the top side (i.e. the side closer to UV lamp during synthesis) of the sheet, (c) and (d) shows the bottom side of the sheet. The small particles on the bottom side were spherical and had a diameter of approximately 2 to 6 μm. The large particles at the top side ranged from approximately 12 to 88 μm and were not spherical in some cases. It can also be seen from micrograph (a) that the large particles are only in the top layer of the sheet. Figure S4: replicates were produced with curing time of 65 s. Figure S5: Measured wetting times of channels with different widths for screen printed materials printed from slurries with different concentrations. Higher concentration of the slurry leads to formation of a material that has a lower wetting time. Figure S6: Screen printed materials with different slurry concentrations after the wetting test. Concentration of the slurry were 0.35, 0.4, 0.43, 0.47, 0.51 g/mL from left to right. Figure S7: Replicate printings of pattern (B) with 0.51 g/mL slurry using screen printing. Figure S8: SEM micrographs of screen printed material. Figure S9: Wetting of replicate Figure 1 (R1) and Figure 1 (R5) produced by direct write printing. Liquid does not wet the leftmost channel in print (R1) and the flow stops for all 3 channels for print (R2). Figure S10: Microscope images of gaps in the channel of direct write printing replicate print R2 (narrowest, middle and widest channel from left to right). Water with blue food colouring has not moved any further from these points since there is a visible large gap between the particles in the channel. Figure S11: SEM pictures of silica gel particles used with direct write printing. The particles were irregularly shaped and range from approximately 66 to 310 μm in size. The inner diameter of 18G needle is 0.838 mm. Figure S12: SEM micrographs of a channel created by direct write printing. Figure S13: Results of printing (with direct write printing) the pattern twice on the same glass slide. Table S1: Energy dispersive x-ray analysis was made with the scanning electron microscope to a sample of the polymer monolith material immediately after synthesis (Day 0) and after 1 week (Day 7). The material was left to stand in the lab at normal conditions for the 7-day period. 4 replicate measurements were made on each day. The weight percent of oxygen significantly increases between Day 0 and Day 7, indicating that oxidation takes place which can lead to formation of polar surface groups. Table S2: Channel widths measured for screen printed slurry with different concentrations. Table S3: Thickness of the screen printed materials where the printed slurry had different concentration. Table S4: Wetting times of the screen printed materials where the printed slurry had different concentration. Table S5: Table with material thickness data of replicates from different printing methods: photolithography (PL), screen printing (SP), direct write printing (DWP). Table S6: Measurement data of channel widths of replicate chips printed with different methods. Table S7: Overall channel width data of replicate chips. Average, standard deviation and relative standard deviation is calculated over the specific channel type for different printing methods. Accuracy is calculated by subtracting designed width from Average value. Table S8: Measurement data of wetting times of chips printed with different methods and filter paper. In one of the replicates for PL, the rectangular shape of the monolith had partly delaminated from the glass slide. This led to a 2.3 to 3.3 time decrease in wetting time. The variance in wetting time between channels of the same chip and between different chips may therefore be also caused by the difference in the degree of delamination of the material from the glass slides. If the replicate with the delaminated part was not taken into account, the average wetting time was 78.1 s/2 cm (RSD 18.1%). Table S9: Overall data on wetting times. Average, standard deviation and relative standard deviation is calculated over the specific channel type for different printing methods.

## References

[1] Culbertson C.T., Mickleburgh T.G., Stewart-James S.A., Sellens K.A., Pressnall M. (2014). Micro Total Analysis Systems: Fundamental Advances and Biological Applications. Anal. Chem..

[2] Jayamohan H., Romanov V., Li H., Son J., Samuel R., Nelson J., Gale B.K. (2017). Advances in Microfluidics and Lab-on-a-Chip Technologies. Molecular Diagnostics.

[3] Gulliksen A., Keegan H., Martin C., O’Leary J., Solli L.A., Falang I.M., Grønn P., Karlgård A., Mielnik M.M., Johansen I.-R. Towards a “Sample-In, Answer-Out” Point-of-Care Platform for Nucleic Acid Extraction and Amplification: Using an HPV E6/E7 MRNA Model System. https://www.hindawi.com/journals/jo/2012/905024/.

[4] Yin J., Zou Z., Hu Z., Zhang S., Zhang F., Wang B., Lv S., Mu Y.A. (2020). “Sample-in-Multiplex-Digital-Answer-out” Chip for Fast Detection of Pathogens. Lab Chip.

[5] Roux D.L., Root B.E., Hickey J.A., Scott O.N., Tsuei A., Li J., Saul D.J., Chassagne L., Landers J.P., De Mazancourt P. (2014). An Integrated Sample-in-Answer-out Microfluidic Chip for Rapid Human Identification by STR Analysis. Lab Chip.

[6] Chin C.D., Linder V., Sia S.K. (2012). Commercialization of Microfluidic Point-of-Care Diagnostic Devices. Lab Chip.

[7] Yetisen A.K., Akram M.S., Lowe C.R. (2013). Paper-Based Microfluidic Point-of-Care Diagnostic Devices. Lab Chip.

[8] Becker H., Gärtner C. (2008). Polymer Microfabrication Technologies for Microfluidic Systems. Anal. Bioanal. Chem..

[9] Sackmann E.K., Fulton A.L., Beebe D.J. (2014). The Present and Future Role of Microfluidics in Biomedical Research. Nature.

[10] Li F., Macdonald N.P., Guijt R.M., Breadmore M.C. (2018). Increasing the Functionalities of 3D Printed Microchemical Devices by Single Material, Multimaterial, and Print-Pause-Print 3D Printing. Lab Chip.

[11] Martinez A.W., Phillips S.T., Butte M.J., Whitesides G.M. (2007). Patterned Paper as a Platform for Inexpensive, Low-Volume, Portable Bioassays. Angew. Chem. Int. Ed..

[12] Santhiago M., Nery E.W., Santos G.P., Kubota L.T. (2014). Microfluidic Paper-Based Devices for Bioanalytical Applications. Bioanalysis.

[13] Cate D.M., Adkins J.A., Mettakoonpitak J., Henry C.S. (2015). Recent Developments in Paper-Based Microfluidic Devices. Anal. Chem..

[14] Li X., Ballerini D.R., Shen W. (2012). A Perspective on Paper-Based Microfluidics: Current Status and Future Trends. Biomicrofluidics.

[15] Carrilho E., Martinez A.W., Whitesides G.M. (2009). Understanding Wax Printing: A Simple Micropatterning Process for Paper-Based Microfluidics. Anal. Chem..

[16] Liu J., Kong X., Wang H., Zhang Y., Fan Y. (2019). Roll-to-Roll Wax Transfer for Rapid and Batch Fabrication of Paper-Based Microfluidics. Microfluid. Nanofluid.

[17] Hu J., Wang S., Wang L., Li F., Pingguan-Murphy B., Lu T.J., Xu F. (2014). Advances in Paper-Based Point-of-Care Diagnostics. Biosens. Bioelectron..

[18] Busa L., Mohammadi S., Maeki M., Ishida A., Tani H., Tokeshi M. (2016). Advances in Microfluidic Paper-Based Analytical Devices for Food and Water Analysis. Micromachines.

[19] Meredith N.A., Quinn C., Cate D.M., Reilly T.H., Volckens J., Henry C.S. (2016). Paper-Based Analytical Devices for Environmental Analysis. Analyst.

[20] De Araujo W.R., Cardoso T.M.G., da Rocha R.G., Santana M.H.P., Muñoz R.A.A., Richter E.M., Paixão T.R.L.C., Coltro W.K.T. (2018). Portable Analytical Platforms for Forensic Chemistry: A Review. Anal. Chim. Acta.

[21] Cummins B.M., Chinthapatla R., Ligler F.S., Walker G.M. (2017). Time-Dependent Model for Fluid Flow in Porous Materials with Multiple Pore Sizes. Anal. Chem..

[22] Lim H., Jafry A.T., Lee J. (2019). Fabrication, Flow Control, and Applications of Microfluidic Paper-Based Analytical Devices. Molecules.

[23] Yamada K., Henares T.G., Suzuki K., Citterio D. (2015). Paper-Based Inkjet-Printed Microfluidic Analytical Devices. Angew. Chem. Int. Ed..

[24] Fichou D., Morlock G.E. (2017). Open-Source-Based 3D Printing of Thin Silica Gel Layers in Planar Chromatography. Anal. Chem..

[25] Fan H., Lu Y., Stump A., Reed S.T., Baer T., Schunk R., Perez-Luna V., López G.P., Brinker C.J. (2000). Rapid Prototyping of Patterned Functional Nanostructures. Nature.

[26] Yuan X., Oleschuk R.D. (2018). Advances in Microchip Liquid Chromatography. Anal. Chem..

[27] Vangelooven J., Malsche W.D., Beeck J.O.D., Eghbali H., Gardeniers H., Desmet G. (2010). Design and Evaluation of Flow Distributors for Microfabricated Pillar Array Columns. Lab Chip.

[28] Merck Millipore (2013). Rapid Lateral Flow Test Strips Considerations for Product Development.

[29] Guiochon G. (2007). Monolithic Columns in High-Performance Liquid Chromatography. J. Chromatogr. A.

[30] Hillmering M., Pardon G., Vastesson A., Supekar O., Carlborg C.F., Brandner B.D., van der Wijngaart W., Haraldsson T. (2016). Off-Stoichiometry Improves the Photostructuring of Thiol–Enes through Diffusion-Induced Monomer Depletion. Microsyst. Nanoeng..

[31] Chen J.-J., Struk K.N., Brennan A.B. (2011). Surface Modification of Silicate Glass Using 3-(Mercaptopropyl)Trimethoxysilane for Thiol–Ene Polymerization. Langmuir.

[32] Banks C.E., Foster C.W., Kadara R.O. (2016). Screen-Printing Electrochemical Architectures.

[33] Karni J., Karni E. (1995). Gypsum in Construction: Origin and Properties. Mater. Struct..

[34] Nimmo J.R., Park M. (2004). Porosity and Pore Size Distribution. Encycl. Soils Environ..

[35] Whatman^®^ Qualitative Filter Paper, Grade 1 WHA1001025. https://www.sigmaaldrich.com/catalog/product/aldrich/wha1001025.

[36] He Y., Wu Y., Fu J.-Z., Wu W.-B. (2015). Fabrication of Paper-Based Microfluidic Analysis Devices: A Review. RSC Adv..

[37] Yamada K., Shibata H., Suzuki K., Citterio D. (2017). Toward Practical Application of Paper-Based Microfluidics for Medical Diagnostics: State-of-the-Art and Challenges. Lab Chip.

[38] Ribeiro L.F., Masini J.C., Svec F. (2019). Use of Thiol Functionalities for the Preparation of Porous Monolithic Structures and Modulation of Their Surface Chemistry: A Review. TrAC Trends Anal. Chem..

[39] Lafleur J.P., Kwapiszewski R., Jensen T.G., Kutter J.P. (2013). Rapid Photochemical Surface Patterning of Proteins in Thiol–Ene Based Microfluidic Devices. Analyst.

[40] Tong S., Liu S., Wang H., Jia Q. (2014). Recent Advances of Polymer Monolithic Columns Functionalized with Micro/Nanomaterials: Synthesis and Application. Chromatographia.

