# Supplementary material for: A New Direction in Microfluidics: Printed Porous Materials
Source: Micromachines (Basel). 2021 Jun 8;12(6):671. doi: 10.3390/mi12060671 (PMC8229541; doi:10.3390/mi12060671)
Supplement: Supplementary file 1 [file micromachines-12-00671-s001.zip › micromachines-1232644-supplementary.pdf]

Article

A New Direction in Microfluidics: Printed Porous Materials

Hanno Evard, Hans Priks, Indrek Saar, Heili Aavola, Tarmo Tamm, Ivo Leito

Photolithography

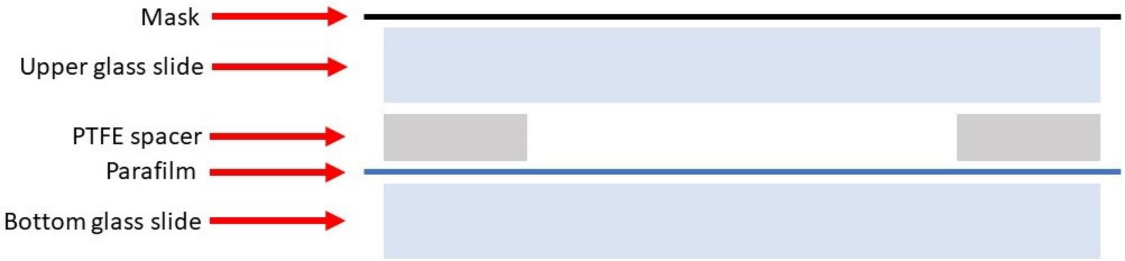

Figure S1. Side view of scheme of photolithography setup. The different layers are held together by paper clamps.

Comments on curing time

If too short curing duration was used (45 seconds), then the channels did not form properly (Figure S1(R1)) because the polymer did not have enough time to cure in all areas. In the case of too long curing time (80 seconds), there was a loss of accuracy (Figure S1(R3)). There can be different causes to loss of accuracy, possibly acting in combination: some UV radiation can pass through the mask, collimated light was not used and therefore UV can pass under the mask, reactive free radicals created during curing can diffuse under the mask [1].

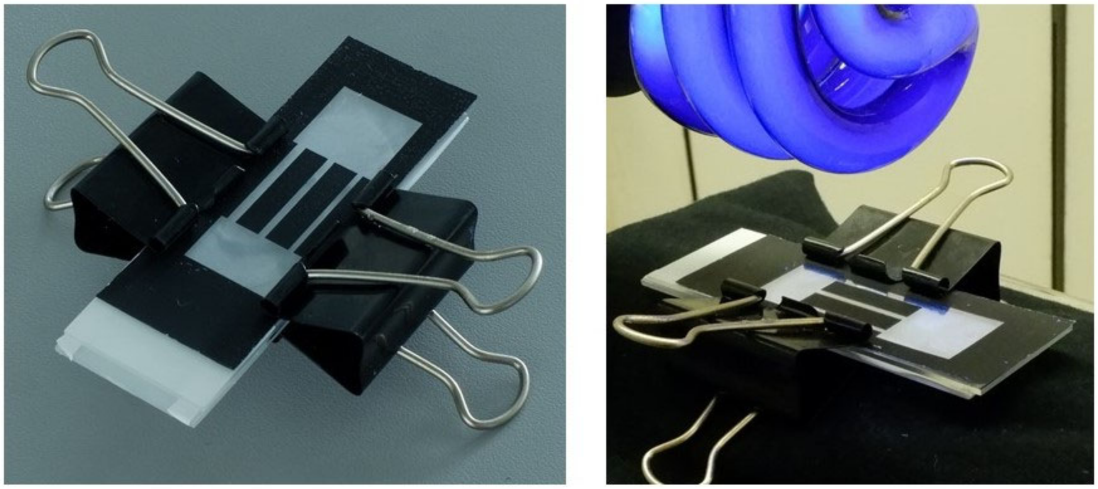

Figure S2. Pictures of photolithography setup.

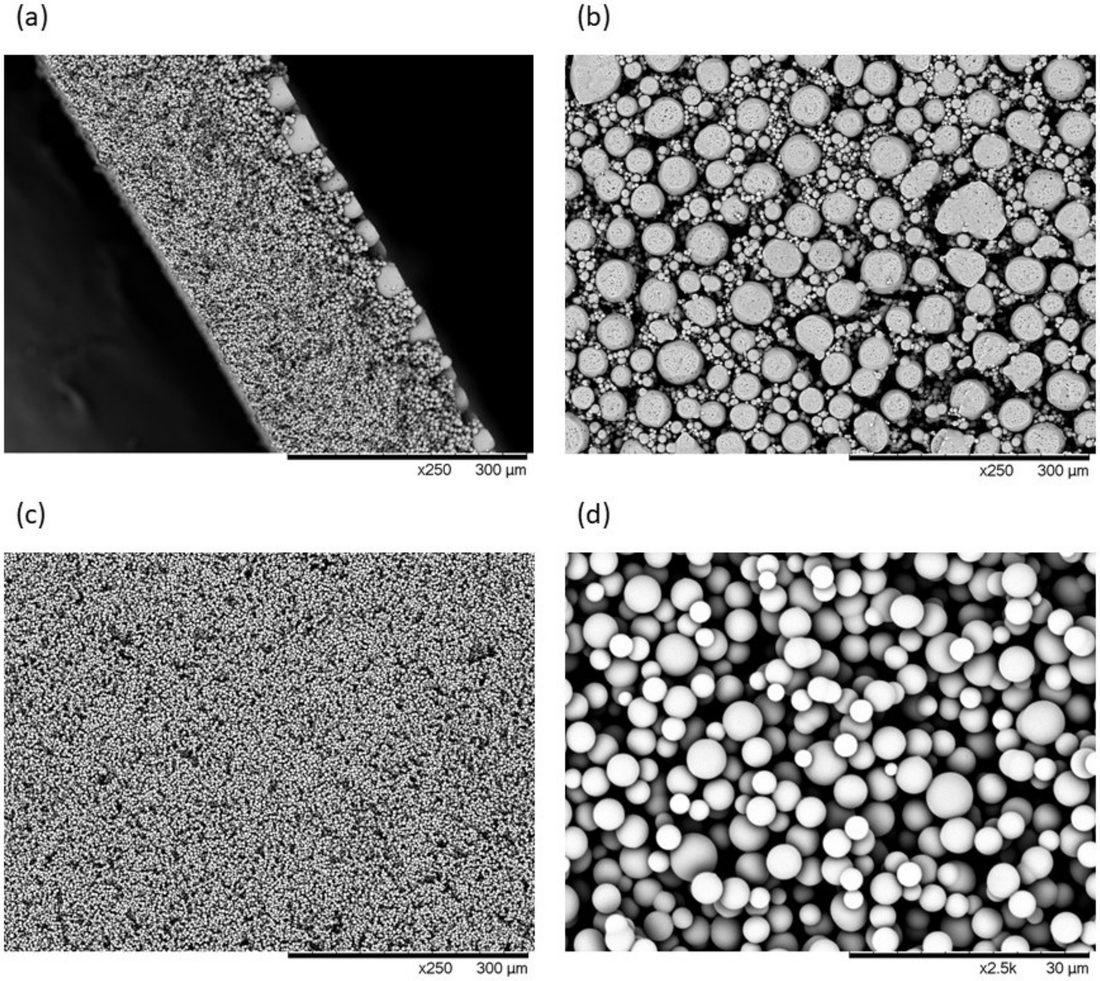

Figure S3. SEM micrographs of the porous structure of the monolithic thiol-ene polymer sheet. Micrograph (a) shows the cross section of the polymer sheet, (b) shows the top side (i.e. the side closer to UV lamp during synthesis) of the sheet, (c) and (d) shows the bottom side of the sheet. The small particles on the bottom side were spherical and had a diameter of approximately 2 to 6 μm. The large particles at the top side ranged from approximately 12 to 88 μm and were not spherical in some cases. It can also be seen from micrograph (a) that the large particles are only in the top layer of the sheet.

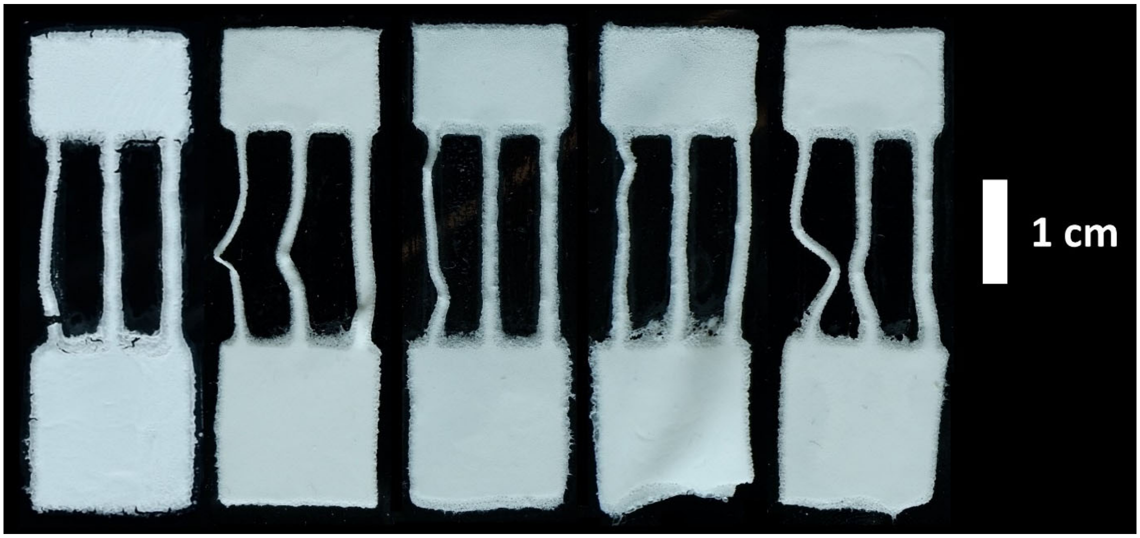

Figure S4. replicates were produced with curing time of 65 second.

Table S1. Energy dispersive x-ray analysis was made with the scanning electron microscope to a sample of the polymer monolith material immediately after synthesis (Day 0) and after 1 week (Day 7). The material was left to stand in the lab at normal conditions for the 7-day period. 4 replicate measurements were made on each day. The weight percent of oxygen significantly increases between Day 0 and Day 7, indicating that oxidation takes place which can lead to formation of polar surface groups.

		Replicate measurements, weight %					
	**Element**	**1**	2	3	4	Average	Stdev
Day 0	Carbon	65.742	65.638	65.544	65.06	65.50	0.30
	Oxygen	14.832	14.964	14.861	15.078	14.93	0.11
	Sulphur	19.426	19.398	19.596	19.862	19.57	0.21
Day 7	Carbon	61.254	62.559	60.906	64.333	62.26	1.55
	Oxygen	22.315	21.997	22.57	22.446	22.33	0.25
	Sulphur	16.43	15.444	16.524	13.22	15.40	1.54

Screen printing

Discussion on accuracy of screen printing with different concentration slurries

Also, the widest (2.4 mm) channel was found to be narrower than the middle (2.1 mm) channel. This could be due to the rheopectic effect of gypsum slurry (i.e. the slurry becomes more viscous during printing). All the prints with SP were performed so that the printing direction was from the narrower channel toward the wider channel (i.e. the narrowest channels were printed before the wider ones). Therefore, as the widest channel was printed, the slurry was more viscous (less ink is printed through the screen if the ink is more viscous). A similar effect can be seen in prints (R6) and (R12) where the wider channel side of the print has significantly less material.

Table S2. Channel widths measured for screen printed slurry with different concentrations

**C (g/ml)**	**Designed width, mm**	**Measured widths in mm** 1	**2**	**3**	4	5	6	Average	Stdev	Relative stdev
0.35	2.4	3.27	2.2	3.65	2.58	3.06	3.33	3.02	0.53	17.7%
	2.1	3.23	3.92	3.35	4.24	4.34	3.75	3.81	0.45	11.9%
	1.8	3.35	2.22	2.16	2.94	3.33	3.00	2.83	0.53	18.6%
0.4	2.4	3.09	3.11	2.28	3.80	2.28	3.70	3.04	0.66	21.7%
	2.1	3.60	3.20	3.91	4.58	3.72	3.34	3.73	0.49	13.2%
	1.8	2.15	3.37	3.12	2.51	2.80	2.57	2.75	0.44	16.0%

0.43	2.4	2.05	2.20	1.86	2.24	1.96	2.61	2.15	0.27	12.3%
	2.1	2.01	2.57	2.34	2.43	2.49	2.51	2.39	0.20	8.5%
	1.8	2.28	1.80	1.67	1.97	1.82	2.26	1.97	0.25	12.9%
0.47	2.4	1.98	1.69	1.83	2.23	2.58	1.88	2.03	0.32	15.9%
	2.1	2.62	2.21	2.52	3.21	2.60	2.90	2.68	0.34	12.8%
	1.8	1.83	2.67	2.54	2.58	2.56	2.37	2.43	0.31	12.7%
0.51	2.4	2.12	1.56	1.98	1.73	2.00	2.27	1.94	0.26	13.3%
	2.1	2.13	1.81	1.69	1.87	2.12	1.85	1.91	0.18	9.2%
	1.8	1.82	1.02	2.03	1.32	1.19	2.13	1.59	0.47	29.5%

Table S3. Thickness of the screen printed materials where the printed slurry had different concentration.

**Article Figure S3**	**C (g/ml)**	**Replicate thickness measurements, mm** 1	2	3	4	5	Average	Stdev	Relative stdev	Overall average	Overall stdev	Overall relative stdev
(R1)	0.35	0.28	0.28	0.32	0.33	0.30	0.30	0.0228	7.6%			
(R2)	0.4	0.33	0.34	0.33	0.33	0.32	0.33	0.0071	2.1%			
(R3)	0.43	0.30	0.30	0.31	0.31	0.30	0.30	0.0055	1.8%	0.339	0.041	12%
(R4)	0.47	0.32	0.32	0.32	0.31	0.32	0.32	0.0045	1.4%			
(R5)	0.51	0.37	0.36	0.37	0.37	0.37	0.37	0.0045	1.2%			
(R6)	0.57	0.42	0.42	0.41	0.41	0.40	0.41	0.0084	2.0%			

Table S4. Wetting times of the screen printed materials where the printed slurry had different concentration.

Article Figure S3	C (g/ml)	**Designed** width, mm	sec	mm	Wetting time (sec / 2 cm)	Average for chip	Stdev for chip	Relative stdev for chip	Overall average	Overall stdev	Overall relative stdev
		2.4	72	18	80.0						
(R1)	0.35	2.1	72	18	80.0	83.6	6.2	7.5%			
		1.8	84	18.5	90.8						
		2.4	76	17.5	86.9						
(R2)	0.4	2.1	76	17.5	86.9	94.5	13.2	14.0%			
		1.8	96	17.5	109.7						
		2.4	100	19	105.3						
(R3)	0.43	2.1	100	18.5	108.1	114.1	12.9	11.3%	140	82	59%
		1.8	116	18	128.9						
		2.4	132	18.5	142.7						
(R4)	0.47	2.1	112	17.5	128.0	143.6	16.0	11.2%			
		1.8	144	18	160.0						
		2.4	196	19.5	201.0						
(R5)	0.51	2.1	160	17.5	182.9	262.7	122.9	46.8%			
		1.8	384	19	404.2						

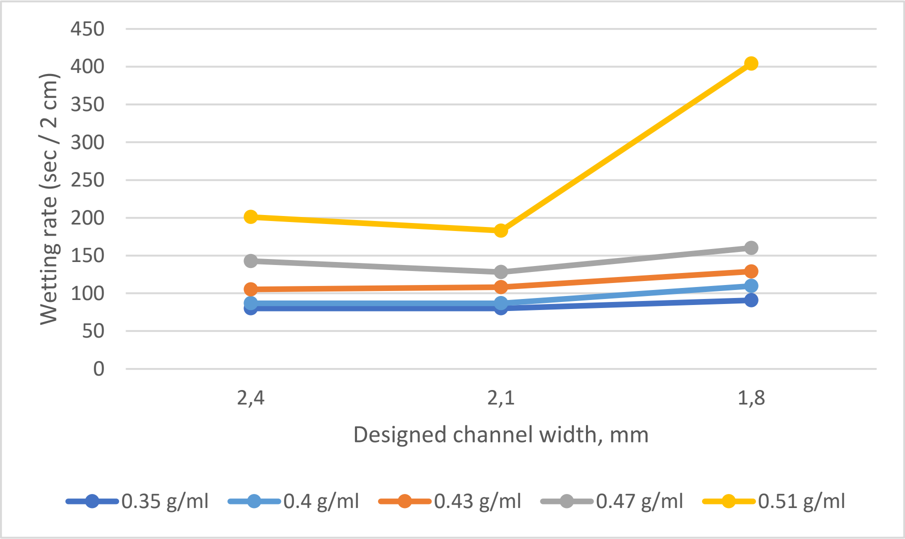

Figure S5. Measured wetting times of channels with different widths for screen printed materials printed from slurries with different concentrations. Higher concentration of the slurry leads to formation of a material that has a lower wetting time.

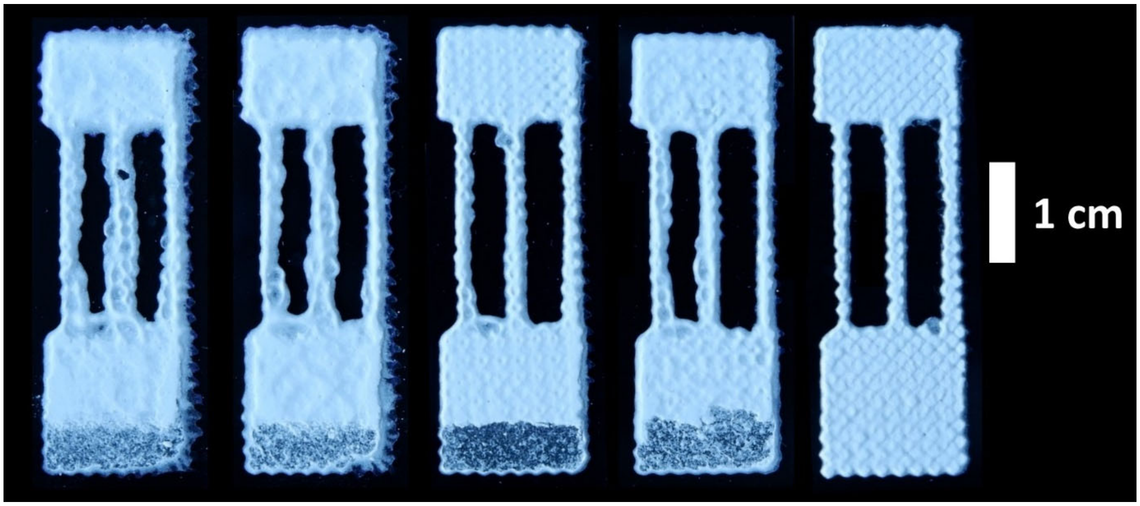

Figure S6. Screen printed materials with different slurry concentrations after the wetting test. Concentration of the slurry were 0.35, 0.4, 0.43, 0.47, 0.51 g/ml from left to right.

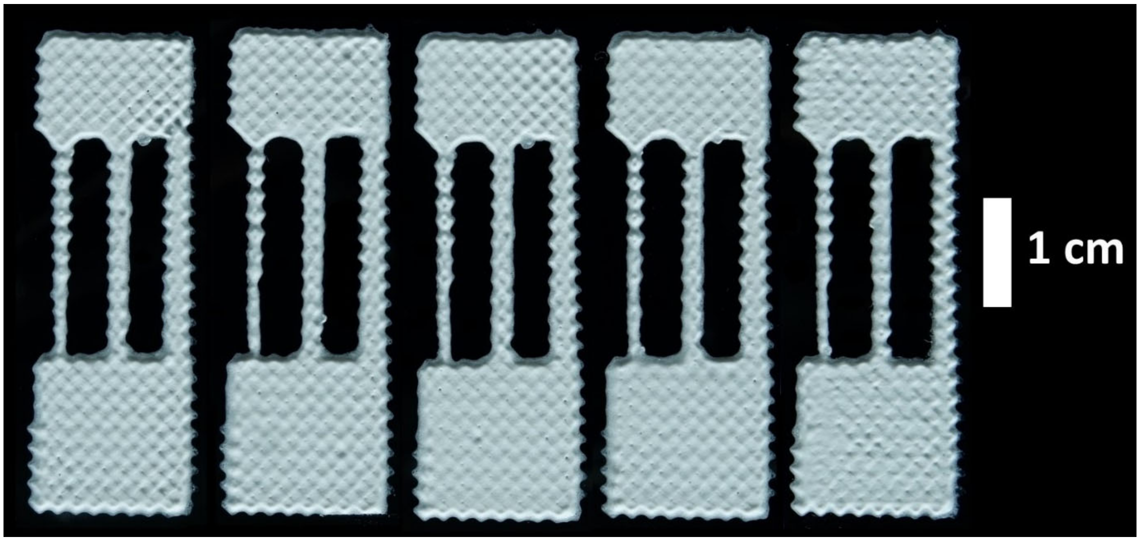

Figure S7. Replicate printings of pattern (B) with 0.51 g/ml slurry using screen printing.

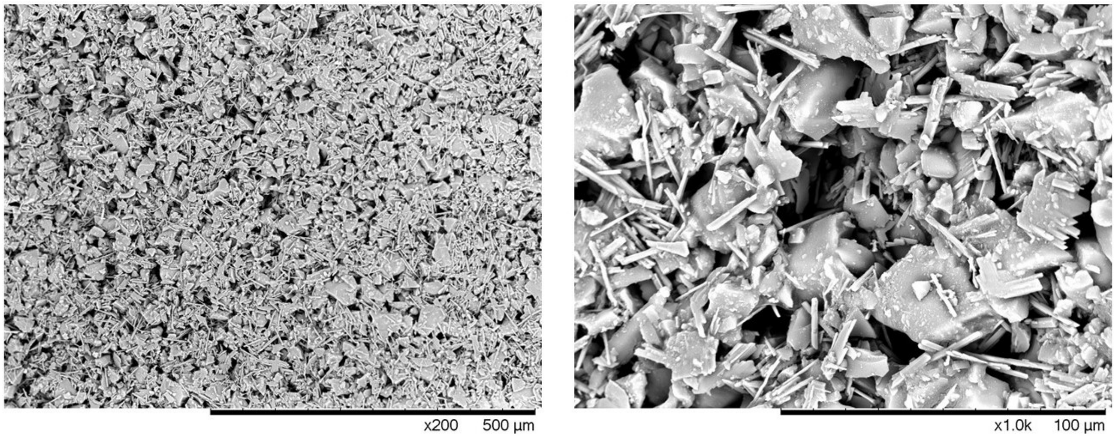

Figure S8. SEM micrographs of screen printed material.

Direct write printing

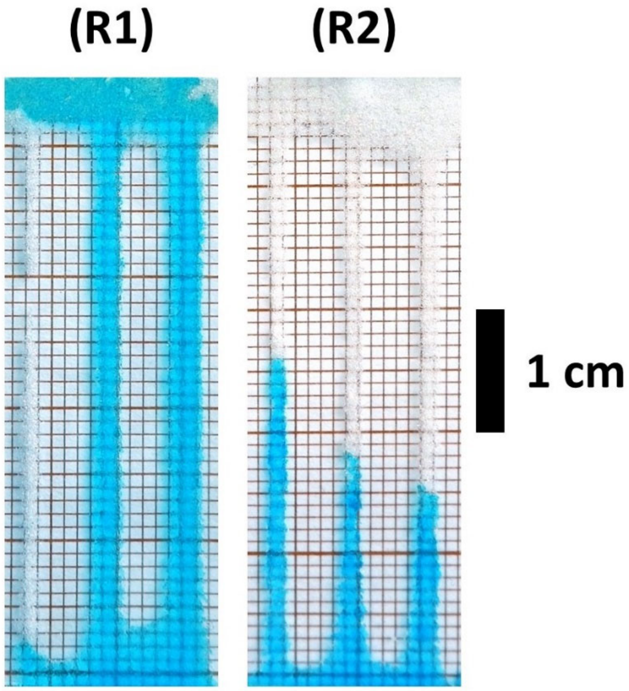

Figure S9. Wetting of replicate Figure S1 (R1) and Figure S1 (R5) produced by direct write printing. Liquid does not wet the leftmost channel in print (R1) and the flow stops for all 3 channels for print (R2).

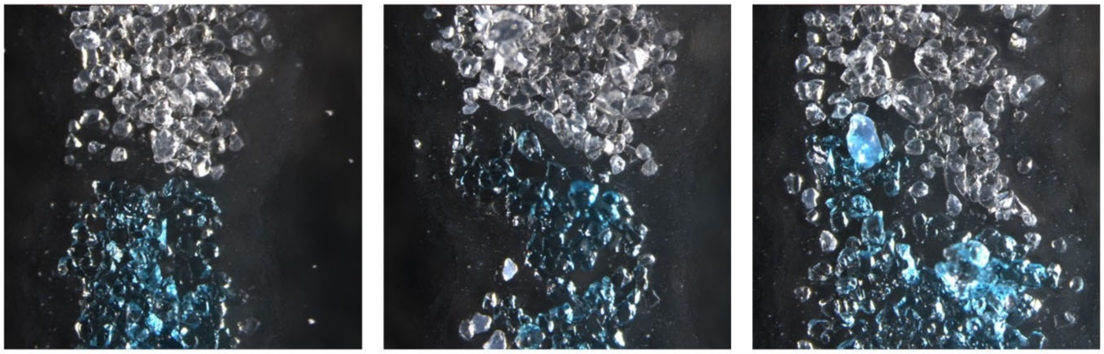

Figure S10. Microscope images of gaps in the channel of direct write printing replicate print R2 (narrowest, middle and widest channel from left to right). Water with blue food colouring has not moved any further from these points since there is a visible large gap between the particles in the channel.

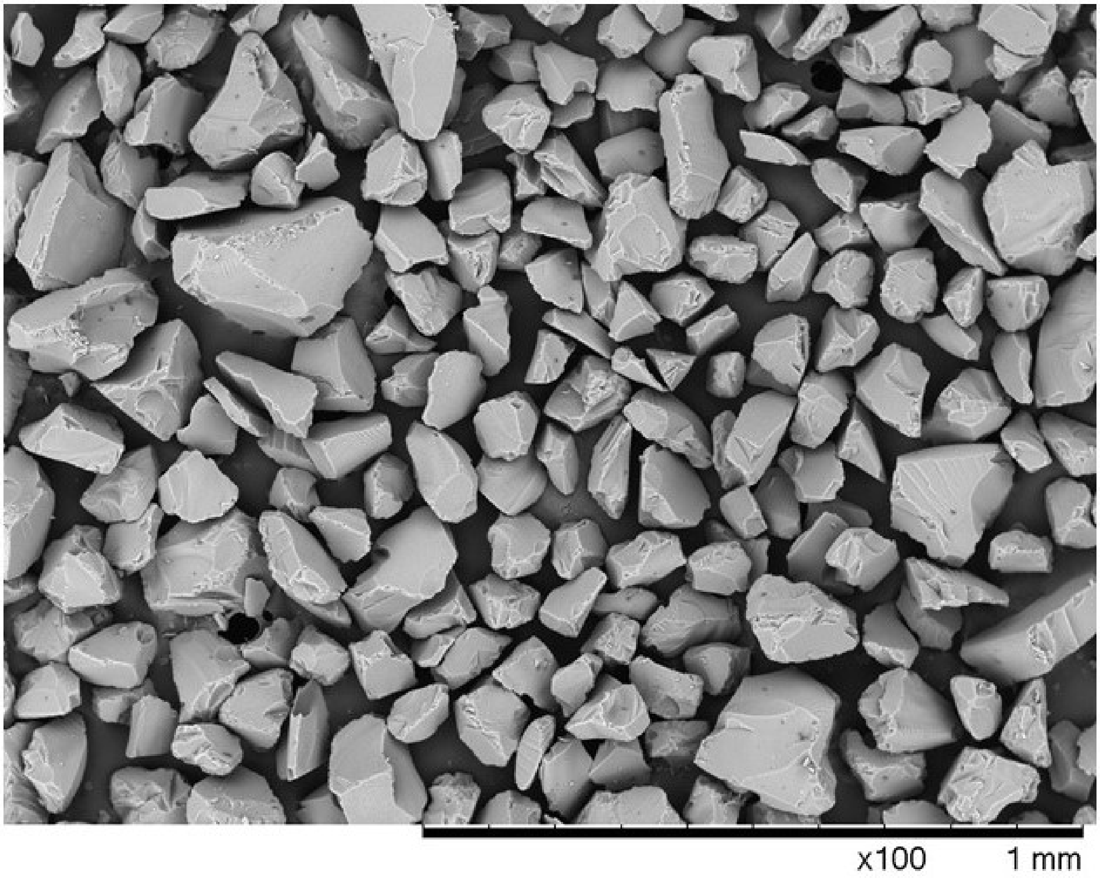

Figure S11. SEM pictures of silica gel particles used with direct write printing. The particles were irregularly shaped and range from approximately 66 to 310 μm in size. The inner diameter of 18G needle is 0.838 mm.

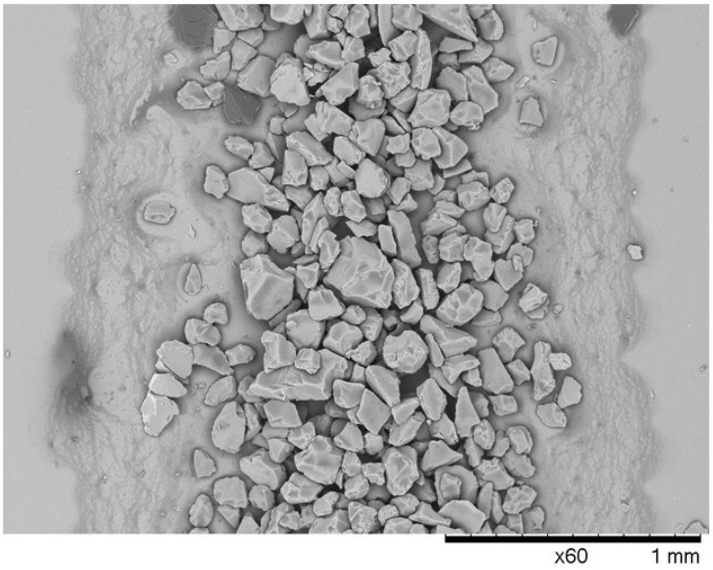

Figure S12. SEM micrographs of a channel created by direct write printing.

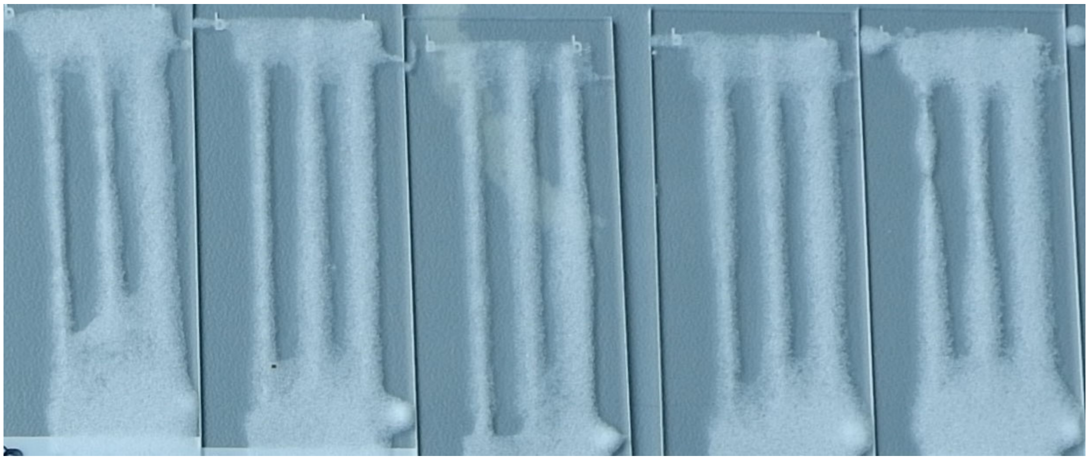

Figure SS13. Results of printing (with direct write printing) the pattern twice on the same glass slide.

Comparison of thickness, width and wetting time of chips formed with three different methods

Table S5. Table Swith material thickness data of replicates from different printing methods: photolithography (PL), screen printing (SP), direct write printing (DWP).

Method	Replicate prints	Replicate thickness measurements, mm 1	2	3	4	5	Average	Stdev	Relative stdev	Overall average	Overall stdev	Overall relative stdev
PL	1	0.30	0.32	0.30	0.31	0.30	0.31	0.009	2.9%	0.31	0.021	6.8%
	2	0.35	0.36	0.32	0.34	0.34	0.34	0.015	4.3%			
	3	0.29	0.33	0.3	0.29	0.30	0.30	0.016	5.4%			
	4	0.30	0.32	0.32	0.31	0.31	0.31	0.008	2.7%			
	5	0.30	0.31	0.28	0.31	0.27	0.29	0.018	6.2%			
SP	1	0.36	0.36	0.37	0.37	0.37	0.37	0.005	1.5%	0.37	0.014	3.8%
	2	0.37	0.39	0.36	0.37	0.36	0.37	0.012	3.3%			
	3	0.38	0.39	0.36	0.36	0.37	0.37	0.013	3.5%			
	4	0.36	0.39	0.36	0.36	0.37	0.37	0.013	3.5%			
	5	0.37	0.41	0.37	0.39	0.40	0.39	0.018	4.6%			
DWP	1	0.42	0.32	0.29	0.28	0.33	0.33	0.055	16.9%	0.33	0.049	14.8%
	2	0.27	0.26	0.26	0.37	0.25	0.28	0.050	17.6%			
	3	0.42	0.37	0.35	0.31	0.36	0.36	0.040	10.9%			
	4	0.38	0.37	0.32	0.33	0.31	0.34	0.031	9.1%			
	5	0.40	0.36	0.32	0.29	0.32	0.34	0.043	12.6%			

Table S6. Measurement data of channel widths of replicate chips printed with different methods.

Method	Replicate	Designed width, mm	Replicate width measurements, mm 1	2	3	4	5	6	Average	Stdev	Relative stdev
PL	1	1.5	2.34	2.04	2.15	2.30	2.31	2.31	2.24	0.12	5.3%
		1.2	2.28	2.06	2.09	2.13	2.14	2.41	2.18	0.13	6.1%
		0.9	1.81	1.57	1.34	1.02	1.39	1.04	1.36	0.30	22.3%
	2	1.5	2.39	2.72	2.42	2.54	2.50	2.15	2.45	0.19	7.7%
		1.2	2.15	1.77	1.79	1.83	1.88	2.15	1.93	0.18	9.1%
		0.9	1.38	0.98	1.09	1.33	1.26	1.47	1.25	0.18	14.7%
	3	1.5	2.45	2.62	2.34	2.53	2.35	2.56	2.48	0.16	4.6%
		1.2	1.86	2.34	1.98	1.83	2.17	1.99	2.03	0.19	9.5%
		0.9	1.83	1.42	1.56	1.34	1.75	1.35	1.54	0.21	13.5%

	4	1.5	2.24	2.53	2.51	2.18	2.24	2.73	2.41	0.22	9.0%
		1.2	1.90	2.05	1.86	2.05	1.94	2.24	2.01	0.14	6.9%
		0.9	1.44	1.70	1.45	1.50	1.33	1.30	1.45	0.15	10.0%
	5	1.5	2.55	2.75	2.12	2.24	2.07	1.96	2.28	0.31	13.4%
		1.2	2.26	2.09	2.02	2.10	2.07	2.10	2.11	0.08	3.8%
		0.9	1.39	1.79	1.44	1.94	1.91	1.82	1.71	0.24	14.1%
SP	1	2.4	3.49	3.70	3.40	3.60	3.48	4.04	3.62	0.23	6.4%
		2.1	2.39	3.19	2.55	2.90	2.68	3.52	2.87	0.42	14.7%
		1.8	1.74	2.07	2.03	2.74	2.36	3.21	2.36	0.54	22.8%
	2	2.4	3.13	3.40	3.30	3.51	4.05	3.41	3.47	0.31	9.1%
		2.1	2.50	3.11	2.87	3.11	2.41	3.13	2.85	0.33	11.4%
		1.8	1.82	2.03	1.83	2.79	1.82	2.92	2.20	0.51	23.4%
	3	2.4	3.24	2.65	3.67	3.10	3.79	3.67	3.35	0.44	13.0%
		2.1	2.36	3.01	2.84	2.63	2.38	2.95	2.70	0.29	10.6%
		1.8	1.66	2.31	1.50	2.65	1.55	2.73	2.07	0.57	27.4%
	4	2.4	2.80	3.34	3.03	3.83	2.80	3.68	3.25	0.44	13.7%
		2.1	2.32	2.91	2.28	2.87	1.91	2.81	2.52	0.41	16.2%
		1.8	1.31	1.85	1.38	2.50	1.64	2.54	1.87	0.54	28.9%
	5	2.4	3.26	3.89	3.57	4.00	3.57	4.35	3.77	0.39	10.2%
		2.1	2.72	3.07	2.83	2.32	3.02	2.43	2.73	0.30	11.1%
		1.8	1.41	1.85	1.42	2.65	1.61	2.58	1.92	0.56	29.3%
DWP	1	widest	3.16	3.07	3.61	2.27	3.26	3.40	3.13	0.46	14.7%
		middle	2.13	2.57	2.41	2.33	2.13	2.35	2.32	0.17	7.3%
		narrowest	1.20	2.05	2.16	1.67	1.01	1.43	1.59	0.46	29.0%
	2	widest	3.00	2.89	2.15	1.90	2.67	2.82	2.57	0.44	17.3%
		middle	2.20	2.40	1.65	1.32	1.49	2.15	1.87	0.44	23.5%
		narrowest	2.34	1.18	1.84	1.41	2.06	1.33	1.69	0.46	27.1%
	3	widest	2.93	3.00	2.86	2.54	3.09	3.31	2.96	0.26	8.7%
		middle	2.71	2.17	2.31	2.49	2.01	2.93	2.44	0.34	14.1%
		narrowest	1.95	2.13	1.55	1.71	1.02	2.46	1.80	0.50	27.7%
	4	widest	3.11	2.76	2.81	3.11	2.59	2.82	2.87	0.21	7.2%
		middle	2.45	1.99	2.13	2.43	2.24	2.68	2.32	0.25	10.7%
		narrowest	1.51	2.13	2.07	1.74	1.32	1.91	1.78	0.32	17.9%
	5	widest	3.34	2.23	3.04	3.26	3.72	3.26	3.14	0.50	15.9%
		middle	2.37	1.98	2.07	2.17	2.21	2.97	2.30	0.36	15.5%
		narrowest	0.69	2.12	1.27	1.51	1.35	2.35	1.55	0.60	39.0%

Table S7. Overall channel width data of replicate chips. Average, standard deviation and relative standard deviation is calculated over the specific channel type for different printing methods. Accuracy is calculated by subtracting designed width from Average value.

Method	Designed width, mm	Average	stdev	Relative stdev	Accuracy
PL	1.5	2.37	0.21	8.8%	0.87
	1.2	2.05	0.16	8.0%	0.85
	0.9	1.46	0.26	17.9%	0.56
SP	2.4	3.49	0.39	11.3%	1.09
	2.1	2.73	0.35	12.9%	0.63
	1.8	2.08	0.54	25.8%	0.28
DWP	widest	2.93	0.42	14.3%	-
	middle	2.25	0.36	16.1%	-
	narrowest	1.68	0.45	27.0%	-

Table S8. Measurement data of wetting times of chips printed with different methods and filter paper. In one of the replicates for PL, the rectangular shape of the monolith had partly delaminated from the glass slide. This led to a 2.3 to 3.3 time decrease in wetting time. The variance in wetting time between channels of the same chip and between different chips may therefore be also caused by the difference in the degree of delamination of the material from the glass slides. If the replicate with the delaminated part was not taken into account, the average wetting time was 78.1 sec / 2 cm (RSD 18.1%).

Method	Replicate	Channel	sec	mm	Wetting time (sec / 2 cm)	Average for chip	Stdev for chip	Relative stdev for chip	Overall average	Overall stdev	Overall relative stdev
PL		1.5	86	19	91				65.4	26.5	40%
	1	1.2	82	19	86	91.5	5.8	6.3%			
		0.9	88	18	98						
		1.5	66	19	69						
	2	1.2	64	18	73	78.8	13.1	16.6%			
		0.9	82	18	94						
		1.5	56	19	61						
	3	1.2	62	18	69	63.9	4.4	6.9%			
		0.9	56	18	62						
		1.5	16	17	19						
	5	1.2	18	17	21	27.5	13.0	47.2%			
		0.9	36	17	42						
SP		2.4	126	20	126				180.1	143.7	80%
	1	2.1	106	18.5	115	209.0	153.7	73.5%			
		1.8	338	17.5	386						
		2.4	112	20	112						
	2	2.1	100	18	111	144.5	57.0	39.5%			
		1.8	184	17.5	210						
		2.4	106	18.5	115						
	3	2.1	104	19	109	124.3	21.4	17.2%			
		1.8	134	18	149						
		2.4	102	18	113						
	4	2.1	100	17.5	114	286.2	298.7	104.3%			
		1.8	426	13.5	631						
		2.4	116	18.5	125						
	5	2.1	116	19	122	136.6	22.3	16.3%			
		1.8	146	18	162						
DWP		widest	36	21	34				35.2	7.3	21%
	R1	middle	36	20	36	35.1	1.2	3.4%			
		narrowest									
		widest	26	18	29						
	R3	middle	26	21	25	28.1	3.0	10.7%			
		narrowest	26	17	31						
		widest	30	18	33						
	R4	middle	30	18	33	32.2	1.9	6.0%			
		narrowest	30	20	30						
		widest	42	17	49						
	R5	middle	42	19	44	45.2	3.8	8.4%			
		narrowest	42	20	42						
	1	2.4	174	20	174	192.7	19.0	9.9%	206.9	24.2	12%

		2.1	192	20	192			
		1.8	212	20	212			
	2	2.4	260	20	260	224.7	30.7	13.7%
		2.1	204	20	204			
		1.8	210	20	210			
Filter paper	3	2.4	192	20	192	196.7	5.0	2.6%
		2.1	196	20	196			
		1.8	202	20	202			
	4	2.4	246	20	246	232.7	16.7	7.2%
		2.1	214	20	214			
		1.8	238	20	238			
	5	2.4	188	20	188	188.0	6.0	3.2%
		2.1	182	20	182			
		1.8	194	20	194			

In case of SP, the largest portion of variance in overall average wetting time comes from slower wetting of the narrowest channels. This can be due to a significant narrowing at the start of a channel, in this case caused by the narrowing in the stencil, leading to lower flow rates over the whole channel [2]. It was also noted that similarly to print (R5), the material did not come off the glass substrate during the wetting test.

Table S9. Overall data on wetting times. Average, standard deviation and relative standard deviation is calculated over the specific channel type for different printing methods.

Method	Channel	Average	Stdev	Relative stdev
PL	1.5	59.8	30.1	50.3%
	1.2	62.4	28.5	45.6%
	0.9	74.0	26.4	35.7%
SP	2.4	118.3	6.9	5.8%
	2.1	114.3	4.9	4.3%
	1.8	226.9	109.5	48.2%
DWP	widest	36.5	8.9	24.5%
	middle	34.1	8.0	23.5%
	narrowest	34.2	6.8	19.8%
Filter paper	2.4	212.0	38.3	18.1%
	2.1	197.6	12.1	6.1%
	1.8	211.2	16.6	7.9%

References

1. Hillmering, M.; Pardon, G.; Vastesson, A.; Supekar, O.; Carlborg, C.F.; Brandner, B.D.; van der Wijngaart, W.; Haraldsson, T. Off-Stoichiometry Improves the Photostructuring of Thiol–Enes through Diffusion-Induced Monomer Depletion. *Microsystems & Nanoengineering* **2016**, *2*, 15043, doi:10.1038/micronano.2015.43.
2. Fu, E.; Ramsey, S.A.; Kauffman, P.; Lutz, B.; Yager, P. Transport in Two-Dimensional Paper Networks. *Microfluidics and nanofluidics* **2011**, *10*, 29–35.
